# The Neutrophil-Platelet Score (NPS) Predicts Survival in Primary Operable Colorectal Cancer and a Variety of Common Cancers

**DOI:** 10.1371/journal.pone.0142159

**Published:** 2015-11-06

**Authors:** David G. Watt, Michael J. Proctor, James H. Park, Paul G. Horgan, Donald C. McMillan

**Affiliations:** Academic Unit of Surgery, School of Medicine–University of Glasgow, Glasgow Royal Infirmary, Glasgow, United Kingdom; Fox Chase Cancer Center, UNITED STATES

## Abstract

**Introduction:**

Recent *in-vitro* studies have suggested that a critical checkpoint early in the inflammatory process involves the interaction between neutrophils and platelets. This confirms the importance of the innate immune system in the elaboration of the systemic inflammatory response. The aim of the present study was to examine whether a combination of the neutrophil and platelet counts were predictive of survival in patients with cancer.

**Methods:**

Patients with histologically proven colorectal cancer who underwent potentially curative resection at a single centre between March 1999 and May 2013 (n = 796) and patients with cancer from the Glasgow Inflammation Outcome Study, who had a blood sample taken between January 2000 and December 2007 (n = 9649) were included in the analysis.

**Results:**

In the colorectal cancer cohort, there were 173 cancer and 135 non-cancer deaths. In patients undergoing elective surgery, cancer-specific survival (CSS) at 5 years ranged from 97% in patients with TNM I disease and NPS = 0 to 57% in patients with TNM III disease and NPS = 2 (p = 0.019) and in patients undergoing elective surgery for node-negative colon cancer from 98% (TNM I, NPS = 0) to 65% (TNM II, NPS = 2) (p = 0.004). In those with a variety of common cancers there were 5218 cancer and 929 non-cancer deaths. On multivariate analysis, adjusting for age and sex and stratified by tumour site, incremental increase in the NPS was significantly associated with poorer CSS (p<0.001).

**Conclusion:**

The neutrophil-platelet score predicted survival in a variety of common cancers and highlights the importance of the innate immune system in patients with cancer.

## Introduction

Colorectal cancer is the second most common cause of cancer death in the UK [1]. In recent decades, mortality rates have been falling and the introduction of bowel screening in many parts of the UK is likely to reduce this further. Despite this, approximately 40% of patients will ultimately die from their disease [2]. In patients with colorectal cancer, surgery remains the main method of cure.

It is now established that the presence of a pre-operative systemic inflammatory response is predictive of disease progression and poorer outcome, regardless of tumour stage, in patients with colorectal cancer [3]. Indeed, systemic inflammation based scoring systems such as the modified Glasgow Prognostic Score (mGPS) and the Neutrophil-Lymphocyte ratio (NLR) have prognostic value in a range of common solid tumours [4–9]. However, with reference to the NLR, multiple thresholds have been used to define high and low NLR values and some have suggested that its prognostic value is mainly derived from the neutrophil count and that the lymphocyte count makes little contribution [10].

Therefore, it is of interest that recent *in-vitro* studies have suggested that a critical checkpoint early in the inflammatory process involves the interaction between neutrophils and platelets [11]. During this process, neutrophils that are recruited to injured tissues/vessels, scan for activated platelets and when detected neutrophils undergo intravascular migration, further elaborating the inflammatory process. This *in-vitro* research highlights the importance of the innate immune system, in particular neutrophils, in the elaboration of the systemic inflammatory response. If the interaction between neutrophils and platelets were of clinical relevance then it might be expected that an elevated neutrophil count in the presence of an elevated platelet count would result in an enhanced systemic inflammatory response. Indeed the combination of a platelet count and the NLR (COP-NLR) has recently been reported as a cumulative predictor of survival in patients with colorectal [12], gastric [13] and oesophageal cancer [14]. However, given that the lymphocyte count makes little contribution to the prognostic value of the NLR [10, 15], it could be hypothesised that a combination of the neutrophil and platelet counts could have considerable prognostic value in predicting survival in patients with cancer. Therefore, the aim of the present study was to examine whether a combination of the neutrophil count and the platelet count was predictive of survival in patients undergoing potentially curative surgery for colorectal cancer and in patients with a variety of common cancers.

## Materials and Methods

For the colorectal cancer cohort, patients with histologically proven colorectal cancer who, on the basis of intra-operative findings and pre-operative computed tomography, were considered to have undergone potentially curative resection at a single centre between March 1999 and May 2013 (n = 813) were initially selected for analysis. Patients in whom a pre-operative neutrophil or platelet count were not available were excluded from analysis (n = 6) as were those patients with TNM stage 0 disease (n = 11). Patient characteristics were collected in a prospectively maintained database and all patient data was anonymised. All tumours were staged according to conventional tumour, node, metastasis classification and additional pathological data obtained from the pathology reports issued at the time of the resection.

Pre-operatively, all patients received thromboembolism prophylaxis and antibiotic prophylaxis as per local protocols and blood samples were taken for routine laboratory analysis. Cut-off values for both neutrophil and platelet count were based on previously reported values [10, 16]. The neutrophil-platelet score (NPS) was calculated as follows Table 1: patients with a neutrophil count ≤7.5 x10^9^/L and platelets ≤400 x10^9^/L scored 0, patients with neutrophils >7.5 x10^9^/L or platelets >400 x10^9^/L scored 1 and patients with both neutrophils >7.5 x10^9^/L and platelets >400 x10^9^/L scored 2.

**Table 1:** The neutrophil platelet score (NPS).

Score	Thresholds
NPS 0	Neutrophils ≤ 7.5 x10^9^/L and Platelets ≤400 x10^9^/L
NPS 1	Neutrophils >7.5 x10^9^/L or Platelets >400 x10^9^/L
NPS 2	Neutrophils >7.5 x10^9^/L and Platelets >400 x10^9^/L

Patients were routinely followed up for 5 years following resection as per national guidelines. Date and cause of death were crosschecked with the cancer registration system and Registrar General (Scotland). Cancer specific survival was measured from date of surgery until date of death. This was an observational study involving patients who underwent surgery for colorectal cancer and their clinical data. As such no specific consent, either written or verbal was obtained to use this data. This was approved by the West of Scotland Research Ethics Committee, Glasgow.

For the larger, common cancer cohort, data was taken from the Glasgow Inflammation Outcome Study [17]. Patients with routine laboratory measurements of C-reactive protein, albumin and a differential white cell count sampled between January 2000 and December 2007, including neutrophil and platelet counts were obtained by systematically searching the North Glasgow biochemical and haematological database systems. Of the 160,481 patients identified, through linkage with the Scottish Cancer Registry using exact matches of the patient’s forename, surname and date of birth, 27 465 were found to have an associated diagnosis of cancer. Of those that had common cancers previously studied in the GIOS cohort, 9649 had been sampled within two years of their cancer diagnosis and were included in the analysis. Cancers were coded according to the International Classification of Disease 10 (ICD-10) and broadly grouped according to the tumour site. Tumours were listed in order of the magnitude of their inflammatory status as previously demonstrated [18]. Patient mortality was established through linkage with the Information Service Division for Scotland (ISD). Patients were excluded if they did not have a blood sample within 2 years of their cancer diagnosis, had incomplete cancer registry follow up, under 16 years old, did not have a complete set of blood results available, had multiple tumours or metastatic disease or had a primary tumour of unknown origin.

### Statistical analysis

The comparison of clinicopathological variables across different NPS scores was performed using a Chi square test. The relationship between the NPS and 5-year survival was examined using log-rank survival analysis. Kaplan-Meier analysis was used to examine the relationship between patients characteristics, NPS, tumour site and cancer-specific and overall survival. Cox proportional hazards multivariate regression models (stratified by tumour site) were used to correct for age and sex and examine the relationship between patient characteristics, NPS and survival. A two-sided p-value of < 0.05 was considered statistically significant. Analyses were performed using SPSS 22.0 (IBM, SPSS, IL, USA).

## Results

A total of 796 patients were included in the analysis of patients undergoing potentially curative surgery for colorectal cancer. The majority were over the age of 65 (66%), male (55%), underwent elective surgery (90%), had an open procedure (87%) and had node negative disease (61%). Median follow up of survivors was 49 (10–180) months with 173 cancer deaths and 135 non-cancer deaths. Table 2 shows the distribution of the clinicopathological characteristics based on the NPS score. Mode of presentation, tumour site, T-stage, TNM stage, margin involvement, peritoneal involvement, tumour perforation and mGPS were significantly different between the 3 NPS groups. On multivariate analysis, adjusting for age and sex and stratified by TNM stage, incremental increase in the NPS was associated with poorer cancer-specific (NPS 1 –HR 1.37, p = 0.091; NPS 2 –HR 1.61, p 0.082) and overall survival (NPS 1 –HR 1.48, p = 0.005; NPS 2 –HR 1.51, p 0.056).

**Table 2:** The relationship between neutrophil platelet score (NPS) and clinicopathological characteristics in patients undergoing potentially curative resection of colorectal cancer.

		All	NPS = 0	NPS = 1	NPS = 2	*p-value*
ClinicopathologicalCharacteristic		n = 796(%)	n = 621(%)	n = 133(%)	n = 42(%)	
**Age**						0.318
	**<65**	266 (34)	210 (34)	42 (32)	14 (33)	
	**65–74**	272 (34)	221 (36)	39 (29)	12 (29)	
	**>75**	258 (32)	190 (30)	52 (39)	16 (38)	
**Sex**						0.553
	**Female**	361 (45)	276 (44)	66 (50)	27 (64)	
	**Male**	435 (55)	345 (56)	67 (50)	23 (55)	
**Presentation**						<0.001
	**Elective**	718 (90)	582 (94)	109 (82)	27 (64)	
	**Emergency**	78 (10)	39 (6)	24 (18)	15 (36)	
**Adjuvant Therapy**						0.241
	**No**	585 (74)	464 (75)	94 (71)	27 (64)	
	**Yes**	211 (26)	157 (25)	39 (29)	15 (36)	
**Tumour Site**						<0.001
	**Colon**	525 (66)	385 (62)	103 (77)	37 (88)	
	**Rectum**	271 (34)	236 (38)	30 (23)	5 (12)	
**T-stage**						<0.001
	**1**	58 (7)	54 (9)	4 (3)	0 (0)	
	**2**	102 (13)	94 (15)	7 (5)	1 (2)	
	**3**	432 (54)	338 (54)	74 (56)	20 (48)	
	**4**	204 (26)	135 (22)	48 (36)	21 (50)	
**N-stage**						0.068
	**0**	486 (61)	391 (63)	71 (53)	24 (57)	
	**1**	224 (28)	172 (28)	42 (32)	10 (24)	
	**2**	86 (11)	58 (9)	20 (15)	8 (19)	
**TNM Stage**						<0.001
	**1**	132 (17)	124 (20)	7 (5)	1 (2)	
	**2**	354 (44)	267 (43)	64 (48)	23 (55)	
	**3**	310 (39)	230 (37)	62 (47)	18 (43)	
**Differentiation**						0.225
	**Mod/well**	710 (90)	560 (91)	113 (86)	37 (88)	
	**Poor**	78 (10)	55 (9)	18 (14)	5 (12)	
**Venous invasion**						0.309
	**No**	354 (45)	285 (46)	53 (40)	16 (38)	
	**Yes**	442 (55)	336 (54)	80 (60)	26 (62)	
**Margin Involvement**						<0.001
	**No**	738 (93)	590 (95)	110 (83)	38 (91)	
	**Yes**	58 (7)	31 (5)	23 (17)	4 (9)	
**Peritoneal Involvement**						0.001
	**No**	617 (78)	499 (80)	93 (70)	25 (60)	
	**Yes**	179 (22)	122 (20)	40 (30)	17 (40)	
**Tumour perforation**						<0.001
	**No**	776 (98)	612 (99)	127 (96)	37 (88)	
	**Yes**	20 (2)	9 (1)	6 (4)	5 (12)	
**Modified Glasgow Prognostic Score (mGPS)**						<0.001
	**0**	505 (63)	450 (73)	46 (35)	9 (21)	
	**1**	164 (21)	102 (16)	45 (34)	17 (41)	
	**2**	127 (16)	69 (11)	42 (31)	16 (38)	
**Survival Status**						<0.001
	**Alive**	488 (61)	407 (66)	64 (48)	17 (40)	
	**Cancer death**	173 (22)	120 (19)	38 (29)	15 (36)	
	**Non-cancer death**	135 (17)	94 (15)	31 (23)	10 (24)	
**Survival (Months)** ^$^		103	107	69	57	<0.001

^$^ median overall survival

Tables 3 and 4 show the relationship between pre-operative NPS, TNM stage and 5 year cancer-specific (CSS) and overall survival (OS). CSS in the whole cohort at 5 years varied from 97% in patients with stage I colorectal cancer to 62% in those with stage III colorectal cancer (p < 0.001) and from 79% in patients with NPS = 0 to 65% in patients with NPS = 2 (p = 0.001). When combined, 5 year CSS varied from 97% in patients with stage I disease and NPS = 0, to 60% in patients with stage III disease and NPS = 2 (p = 0.026). OS at 5 years ranged from 86% in patients with stage I disease to 52% in patients with stage III disease (p < 0.001) and from 68% in patients with NPS = 0 to 48% in patients with NPS = 2 (p < 0.001). When combined, OS at 5 years ranged from 89% in patients with stage I disease and NPS = 0 to 49% in patients with stage III disease and NPS = 2 (p = 0.001).

**Table 3:** The relationship between neutrophil platelet score (NPS) and 5 year cancer-specific survival in patients undergoing curative resection of colorectal cancer. CSS—cancer-specific survival. Survival not calculated if *n*<10.

	NPS = 0(Neut ≤ 7.5 x10^9^/L and Plat ≤400 x10^9^/L)		NPS = 1(Neut >7.5 x10^9^/L or Plat >400 x10^9^/L)		NPS = 2(Neut >7.5 x10^9^/L and Plat >400 x10^9^/L)		All(NPS 0–2)	
All Patients	*n*	5-yr CSS % (SE)	*n*	5-yr CSS % (SE)	*n*	5-yr CSS % (SE)	n	5-yr CSS % (SE)
Stage I	124	97 (2)	7	-	1	-	132	97 (2)
Stage II	267	85 (3)	64	79 (6)	23	68 (11)	354	82 (2)
Stage III	230	63 (4)	62	56 (7)	18	60 (12)	310	62 (3)
All (Stage 0-III)	621	79 (2)	133	69 (5)	42	65 (8)	796	76 (2)
**Elective**	***n***	**5-yr CSS % (SE)**	***n***	**5-yr CSS % (SE)**	***n***	**5-yr CSS % (SE)**	***n***	**5-yr CSS % (SE)**
Stage I	124	97 (2)	7	-	1	-	132	97 (2)
Stage II	248	85 (3)	53	79 (6)	14	68 (13)	315	83 (2)
Stage III	210	65 (4)	49	58 (8)	12	57 (15)	271	63 (3)
All (Stage 0-III)	582	80 (2)	109	70 (5)	27	62 (10)	718	78 (2)
**Elective, Node Negative**	***n***	**5-yr CSS % (SE)**	***n***	**5-yr CSS % (SE)**	***n***	**5-yr CSS % (SE)**	***n***	**5-yr CSS % (SE)**
Stage I	124	97 (2)	7	-	1	-	132	97 (2)
Stage II	248	85 (3)	53	79 (6)	14	68 (13)	315	83 (2)
All (Stage 0-II)	372	89 (2)	60	81 (6)	15	71 (12)	447	87 (2)
**Elective, Node Negative Colon**	***n***	**5-yr CSS % (SE)**	***n***	**5-yr CSS % (SE)**	***n***	**5-yr CSS % (SE)**	***n***	**5-yr CSS % (SE)**
Stage I	71	98 (2)	6	-	0	-	77	99 (1)
Stage II	161	89 (3)	40	82 (7)	12	65 (14)	213	77 (4)
All (Stage 0-II)	232	91 (2)	46	84 (6)	12	65 (14)	290	89 (2)

**Table 4:** The relationship between neutrophil platelet score (NPS) and 5 year overall survival in patients undergoing potentially curative resection of colorectal cancer. OS—overall survival. Survival not calculated if *n*<10.

	NPS = 0(Neut ≤ 7.5 x10^9^/L and Plat ≤400 x10^9^/L)		NPS = 1(Neut >7.5 x10^9^/L or Plat >400 x10^9^/L)		NPS = 2(Neut >7.5 x10^9^/L and Plat >400 x10^9^/L)		All(NPS 0–2)	
All Patients	*n*	5-yr OS % (SE)	*n*	5-yr OS % (SE)	*n*	5-yr OS % (SE)	n	5-yr OS % (SE)
Stage I	124	89 (4)	7	-	1	-	132	86 (4)
Stage II	267	73 (3)	64	68 (6)	23	45 (11)	354	70 (3)
Stage III	230	54 (4)	62	45 (7)	18	49 (12)	310	52 (3)
All (Stage 0-III)	621	68 (2)	133	56 (5)	42	48 (8)	796	65 (2)
**Elective**	***n***	**5-yr OS % (SE)**	***n***	**5-yr OS % (SE)**	***n***	**5-yr OS % (SE)**	***n***	**5-yr OS % (SE)**
Stage I	124	89 (4)	7	-	1	-	132	86 (4)
Stage II	248	74 (3)	53	68 (7)	14	37 (14)	315	71 (3)
Stage III	210	55 (4)	49	45 (8)	12	42 (14)	271	52 (3)
All (Stage 0-III)	582	69 (2)	109	56 (5)	27	41 (10)	718	66 (2)
**Elective, Node Negative**	***n***	**5-yr OS % (SE)**	***n***	**5-yr OS % (SE)**	***n***	**5-yr OS % (SE)**	***n***	**5-yr OS % (SE)**
Stage I	124	89 (4)	7	-	1	-	132	86 (4)
Stage II	248	74 (3)	53	68 (7)	14	37 (14)	315	71 (3)
All (Stage 0-II)	372	78 (3)	60	65 (7)	15	42 (14)	447	75 (2)
**Elective, Node Negative Colon**	***n***	**5-yr OS % (SE)**	***n***	**5-yr OS % (SE)**	***n***	**5-yr OS % (SE)**	***n***	**5-yr OS % (SE)**
Stage I	71	56 (12)	6	-	0	-	77	83 (5)
Stage II	161	75 (4)	40	25 (10)	12	46 (15)	213	72 (4)
All (Stage 0-II)	232	78 (3)	46	24 (9)	12	46 (15)	290	75 (3)

The combination of the platelet count and NLR (COP-NLR) was calculated (using an NLR threshold of 5) in order to determine its effect on survival in patients with operable colorectal cancer. CSS in the whole cohort at 5 years ranged from 78% in patients with COP-NLR = 0 to 67% in patients with COP-NLR = 2 (p = 0.010). Furthermore, on multivariate analysis, adjusting for age and sex and stratified by TNM stage, incremental increase in the COP-NLR was not independently associated with cancer-specific survival (COP-NLR 1 –HR 1.31, p = 0.112; COP-NLR 2 –HR 1.41, p 0.268). Therefore, in comparison to COP-NLR, the NPS was superior in predicting survival in patients with operable colorectal cancer.

As emergency surgery, presence of a colonic tumour and nodal status were associated with the NPS, to control for confounding of these, 5 year survival (both CSS and OS) was examined in patients undergoing elective surgery and then in patients with node negative disease and node negative colonic disease Tables 3 and 4.

In patients undergoing elective surgery, CSS at 5 years ranged from 97% in stage I disease to 63% in stage III disease (p < 0.001) and from 80% in patients with NPS = 0 to 62% in patients with NPS = 2 (p = 0.001). When combined, CSS at 5 years ranged from 97% in patients with stage I disease and NPS = 0 to 57% in patients with stage III disease and NPS = 2 (p = 0.019). A similar relationship was observed in OS at 5 years as survival ranged from 86% to 52% (p < 0.001) and from 69% to 41% (p < 0.001) with TNM stage and NPS alone, the combination of TMN stage and NPS stratified OS from 89% (TNM I, NPS = 0) to 42% (TNM III, NPS = 2) (p < 0.001).

In patients undergoing elective surgery for node negative disease, CSS at 5 years ranged from 97% in stage I disease to 83% in stage II disease (p = 0.003) and from 89% in patients with NPS = 0 to 71% in patients with NPS = 2 (p = 0.002). When combined CSS ranged from 97% (TNM stage I, NPS = 0) to 68% (TNM stage II, NPS = 2) (p = 0.018). Similarly, OS at 5 years ranged from 86% to 71% (p = 0.012) and from 78% to 42% (p < 0.001) with TNM stage and NPS alone and the combination of TNM stage and NPS stratified OS from 89% (TNM I, NPS = 0) to 37% (TNM II, NPS = 0) (p < 0.001).

In patients undergoing elective surgery for node negative colonic disease CSS at 5 years ranged from 99% in stage I disease to 77% in stage II disease (p = 0.003) and from 91% in patients with NPS = 0 to 65% in patients with NPS = 2 (p < 0.001). When combined, CSS ranged from 98% (TNM stage I, NPS = 0) to 65% (TNM stage II, NPS = 2) (p = 0.004). Similarly, OS at 5 years ranged from 83% to 72% (p = 0.039) and from 78% to 46% (p < 0.001) with TNM stage and NPS alone, the combination of TNM stage and NPS stratified OS from 56% (TNM stage I, NPS = 0) to 46% (TNM stage II, NPS = 2) (p = 0.002).

The relationship between the clinicopathological characteristics and the NPS score in patients with a range of common cancers are in shown in Table 5. Age, sex, mode of presentation, tumour site, mGPS, survival status and survival length were significantly different between NPS groups. On multivariate analysis, adjusting for age and sex and stratified by tumour site, incremental increase in the NPS was significantly associated with poorer cancer-specific (NPS 1 –HR 1.60, p < 0.001; NPS 2 –HR 2.14, p < 0.001) and overall survival (NPS 1 –HR 1.61, p < 0.001; NPS 2 –HR 2.19, p < 0.001).

**Table 5:** The relationship between neutrophil platelet score (NPS) and patient demographics in an incidentally sampled cohort of patients with cancer.

		All	NPS = 0	NPS = 1	NPS = 2	*p-value*
		n = 9649(%)	n = 5933(%)	n = 2779(%)	n = 937(%)	
**Age**						<0.001
	**<65**	4631 (48)	3032 (51)	1170 (42)	429 (46)	
	**65–74**	2885 (30)	1696 (29)	886 (32)	303 (32)	
	**>75**	2133 (22)	1205 (20)	723 (26)	205 (22)	
**Sex**						<0.001
	**Female**	4584 (48)	2646 (45)	1468 (53)	470 (50)	
	**Male**	5065 (52)	3287 (55)	1311 (47)	467 (50)	
**Presentation**						<0001
	**Non-Emergency**	6098 (63)	4236 (71)	1398 (50)	464 (50)	
	**Emergency**	3551 (37)	1697 (29)	1381 (50)	473 (50)	
**Tumour Site**						<0.001
	**Breast**	1921 (20)	1611 (27)	268 (10)	42 (5)	
	**Bladder**	437 (4)	259 (4)	128 (5)	50 (5)	
	**Gynaecological**	507 (5)	298 (5)	142 (5)	67 (7)	
	**Prostatic**	509 (5)	322 (6)	159 (6)	28 (3)	
	**Gastroesophageal**	933 (10)	548 (9)	294 (10)	91 (10)	
	**Haematological**	914 (9)	678 (12)	188 (7)	48 (5)	
	**Renal**	459 (5)	288 (5)	134 (5)	37 (4)	
	**Colorectal**	1086 (11)	604 (10)	356 (13)	126 (13)	
	**Head And Neck**	633 (7)	365 (6)	204 (7)	64 (7)	
	**Hepaticopancreaticobiliary**	556 (6)	309 (5)	183 (6)	64 (7)	
	**Pulmonary**	1694 (18)	651 (11)	723 (26)	320 (34)	
**mGPS**						<0.001
	**0**	4013 (42)	3305 (56)	629 (23)	79 (9)	
	**1**	2757 (28)	1504 (25)	931 (33)	322 (34)	
	**2**	2879 (30)	1124 (19)	1219 (44)	536 (57)	
**Survival Status**						<0.001
	**Alive**	3502 (36)	2757 (47)	633 (23)	112 (12)	
	**Cancer death**	5218 (54)	2620 (44)	1849 (66)	749 (80)	
	**Non-cancer death**	929 (10)	556 (9)	297 (11)	76 (8)	
**Survival (Months)** ^$^		21	55	7	3	<0.001

^$^ median overall survival

**Fig 1:**
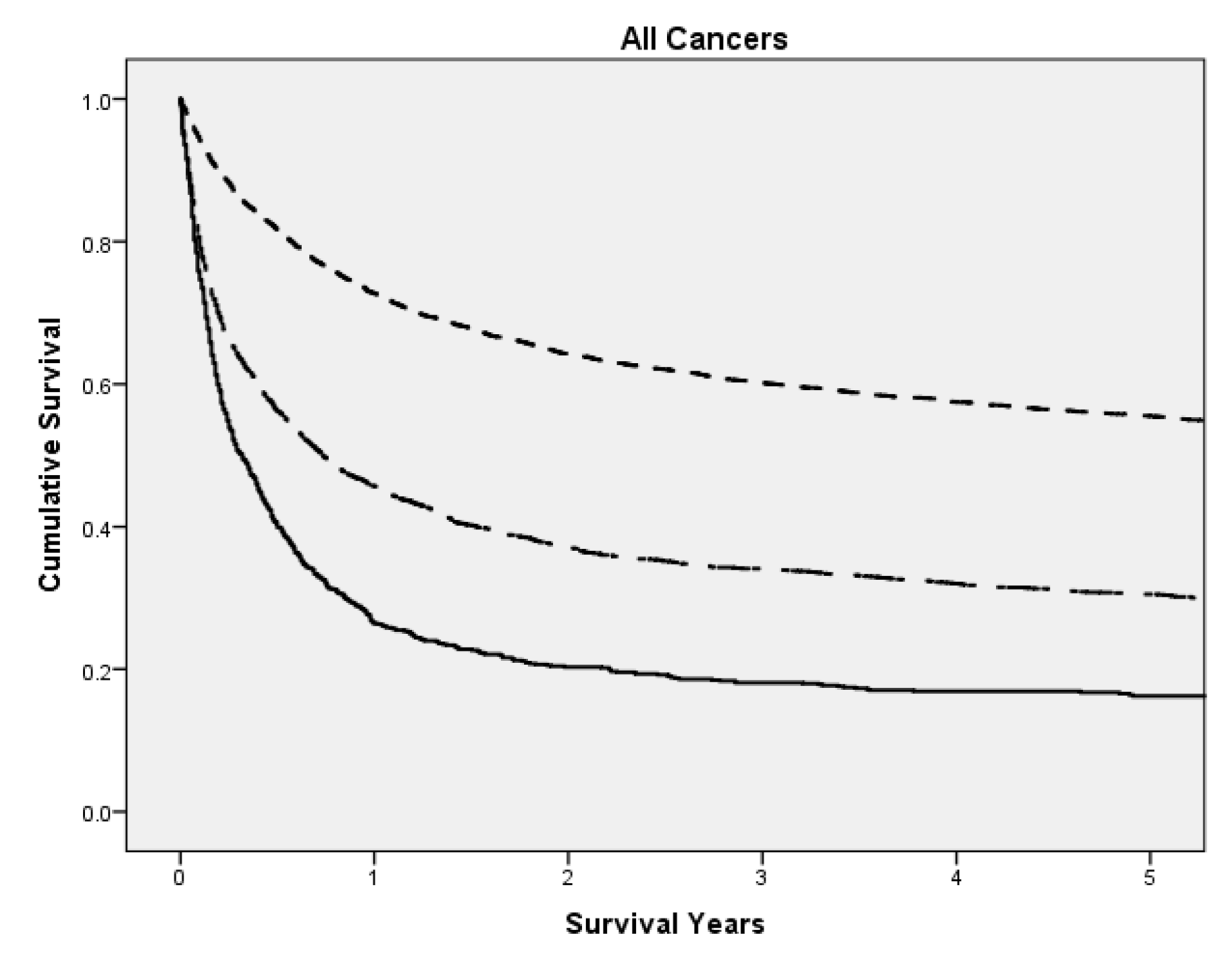
The relationship between the NPS and cancer specific survival in all patients of the GIOS cohort. NPS 0 (top, small dash line), NPS 1 (middle, large dash line) and NPS 2 (bottom, solid line) (p<0.001).

**Fig 2:**
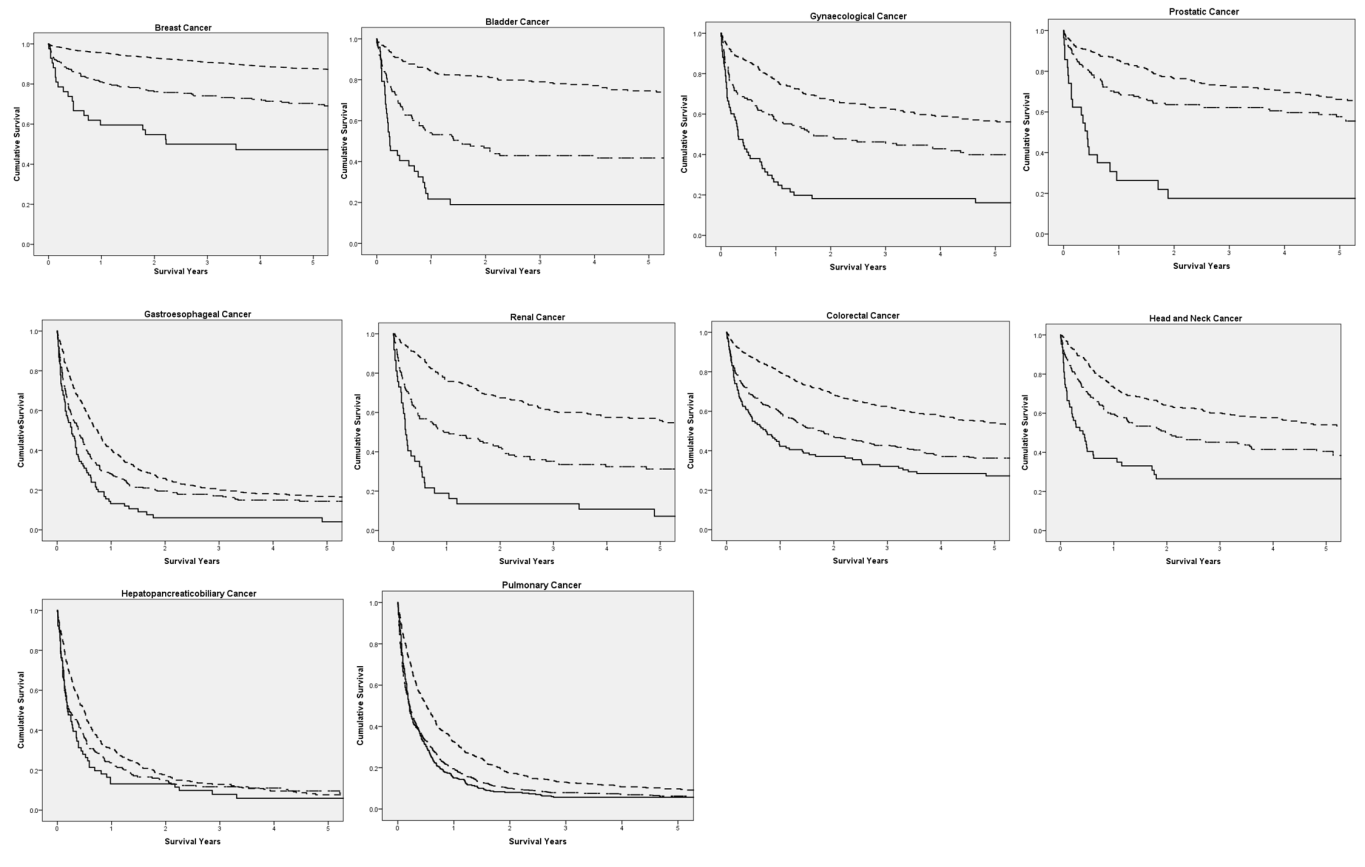
The relationship between the NPS and cancer specific survival in each tumour site. NPS 0 (top, small dash line), NPS 1 (middle, large dash line) and NPS 2 (bottom, solid line).Breast p<0.001, bladder <0.001, gynaecological <0.001, prostatic <0.001, gastroesophageal <0.001, renal <0.001, colorectal <0.001, head and neck <0.001, HPB = 0.009 and pulmonary <0.001.

On Kaplan Meier survival analysis, a greater neutrophil-platelet score is associated with poorer cancer-specific survival in all patients (p < 0.001) Fig 1. On Kaplan Meier survival analysis, based on individual tumour types Fig 2, increasing NPS was significantly associated with poorer cancer-specific survival in patients with breast (p < 0.001), bladder (p < 0.001), colorectal (p < 0.001), gastroeosophageal (p < 0.001), gynaecological (p < 0.001), head and neck (p < 0.001), Hepaticopancreaticobiliary (HPB) (p = 0.009), prostatic (p < 0.001), pulmonary (p < 0.001) and renal cancers (p < 0.001).

## Discussion

The results of the present study show that the combination of neutrophils and platelets in a clinical scoring system, the neutrophil-platelet score (NPS), can be used to predict survival, independent of TNM stage, in patients undergoing potentially curative surgery for colorectal cancer. Furthermore, the results of the present study provide evidence that this simple, novel, objective score has prognostic value in a variety of common cancers. These results confirm the importance of activation of the innate immune response in predicting outcome in patients with cancer.

The results of the present study are consistent with those of Ishizuka and colleagues who reported that the combination of platelets and the NLR was a predictor of post-operative survival in both colorectal and gastric cancer [12, 13]. However, recent evidence would appear to suggest that when using the differential white cell count to predict outcomes, the neutrophil count is the dominant component and as a result the lymphocyte count adds little to its prognostic effect [10]. Furthermore, recent work [12, 13] has suggested that the combination of a platelet count to the NLR (COP-NLR) improves the prediction of outcome. In the present study when the prognostic value of the COP-NLR was examined, the NPS had superior prognostic value. Due to the differences in the formation of the COP-NLR and the NPS the basis of the difference in prognostic value is not clear. Nevertheless, taken together these results would suggest that neutrophils and platelets were the main factors determining the prognostic value of the COP-NLR.

There are other systemic inflammation based scores that have prognostic value in patients with primary operable colorectal cancer and a variety of common solid tumours. The most validated of these is the GPS/mGPS [5, 8, 17]. Indeed, it was of interest in the present study that as the NPS increased from 0 to 2 the median concentration of CRP increased from 6 to 55 mg/L and median concentration of albumin decreased from 38 to 36 g/L *(both p < 0*.*001)*. Therefore it would appear that both these scoring systems are related measures of the systemic inflammatory response. Nevertheless, the present results are of considerable interest since the GPS/mGPS requires the measurement of two acute phase proteins and in many centres they may not be routinely assessed. Together with previous results [19] the present results show the complementary prognostic value of the NPS.

It is of interest that Kumar and colleagues recently reported that, in 1300 patients in phase I clinical cancer trials, the neutrophil-lymphocyte ratio (NLR) was an independent prognostic factor for overall survival [15]. Furthermore, they reported that the neutrophil count but not the lymphocyte count had prognostic value. This finding is consistent with our work in patients with primary operable colorectal cancer [10, 16]. The results of the present study demonstrate that the combination of increased neutrophils and platelets (both components of the innate immune response) was associated with an elaboration of the systemic inflammatory response and significantly poorer survival in patient with a range of common cancers. Taken together, these findings suggest that activation of the innate immune response is a key step in disease progression and poor survival in patients with cancer.

The elaboration of this systemic inflammatory response and the presence of high numbers of neutrophils and platelets may result in an enhancement of cellular breakdown and proliferation (tissue remodelling). Specifically, neutrophils contain multiple enzymes such as, myeloperoxidase, interleukin-6 (IL-6), defensins, lysozyme and collagenase which may directly promote cancer cell intravasation and extravasation [20, 21]. Moreover, activated platelets contain significant quantities of IL-6 and secrete factors such as vascular-endothelial growth factor (VEGF) and other factors that promote angiogenesis and may prevent recognition of cancer cells by the body’s own immune system [22–24]. Furthermore, both neutrophils and platelets are stimulated by IL-6. This may tip the tumour microenvironment towards disease dissemination and promotion of the growth of metastatic disease.

The present study has a number of possible limitations. Detailed data on the use of pre-operative chemo/radiotherapy in the colorectal cancer cohort and its relation to the timing of the pre-operative blood samples was not available. In both cohorts, data relating to other factors that may have affected neutrophil or platelet levels such as drugs and other co-morbidities were not available.

In conclusion, the neutrophil-platelet score can predict survival in patients undergoing potentially curative surgery for colorectal cancer and in a variety of common cancers. This confirms the importance of activation of the innate immune system in patients with cancer.
